# The effects of dance interventions on reducing the risk of falls in older adults: a network meta-analysis

**DOI:** 10.3389/fpubh.2024.1496692

**Published:** 2024-12-16

**Authors:** Ying Li, Zhaoguo Wang, Jiahao Li, Honghao Yang, Zilong Fang

**Affiliations:** ^1^Sports Department, Harbin Engineering University, Harbin, China; ^2^School of Sports Medicine and Rehabilitation, Beijing Sport University, Beijing, China

**Keywords:** dance, older adults, falls, balance, network meta-analysis

## Abstract

**Objective:**

The efficacy of dance in reducing fall risks among older adults highlights its potential for geriatric health, but the optimal dance style remains uncertain. The present study endeavors to systematically analyze the effects of various dance forms on reducing the risk of falls among older adult, with the aim of providing evidence-based insights into the most efficacious dance styles for this population.

**Design:**

The network meta-analysis of the existing literature was conducted to synthesize the available evidence regarding the effectiveness of various dance interventions in reducing the risk of falls among older adults.

**Methods:**

We searched seven databases for randomized controlled trials on dance interventions for fall prevention in older adults and used Stata 17.0 for network meta-analysis.

**Results:**

Twenty-seven studies (*n* = 1,219 older adults) were included. Creative Dance (CD) significantly improved the Berg Balance Scale score in healthy older adults (*p* < 0.05) and ranked best. Folk Dance (FD), CD, and Ballroom Dance (BD) all significantly improved Timed Up and Go time compared to controls, with FD ranking best overall. In Parkinson’s disease patients, both BD and FD significantly improved the Berg Balance Score, with FD again ranking best.

**Conclusion:**

Creative dance, Folk dance, and Ballroom dance effectively reduce the risk of falls in older adults. Creative dance enhanced the Berg Balance Score the most among healthy older adults, while Folk dance improved the Timed Up and Go test performance the best. Similarly, among patients with Parkinson’s disease, Folk dance exhibits the best effect in improving Berg Balance Scale scores.

**Clinical trial registration:**

The protocol of this study was registered with the International Prospective Register of Systematic Review, PROSPERO, under the identification number CRD42022323426, and can be integrally assessed online (https://www.crd.york.ac.uk/prospero/display_record.php?ID=CRD42022323426).

## Introduction

Falls are inadvertent events involving the descent of an individual to the ground or any other lower surface (1). These incidents pose a significant health risk, with over half of falls leading to soft tissue injuries, fractures, and other severe injuries, or even fatalities (2). Furthermore, even in the absence of physical harm, falls can inflict long-lasting psychological sequelae, including depression and an increased fear of falling, which can subsequently limit daily and social activities, deteriorate physical fitness and function, and exacerbate the risk of future falls (3, 4). Alarmingly, approximately 28 to 35% of individuals aged 65 and older experience a fall annually, with this proportion escalating to 32 to 42% among those aged 70 and above (5). These statistics highlight the pressing need for effective interventions to mitigate the burden of falls on older adults.

Risk factors for falls can be categorized into intrinsic and extrinsic categories. Intrinsic factors encompass aging, chronic illnesses, reduced muscle strength, gait and balance disorders, as well as cognitive impairments. Conversely, extrinsic factors comprise medication use, unfavorable environments, and hazardous activities (6). Notably, among these factors, diminished balance ability stands out as a primary cause of falls among older adults (7). Specifically, the balance ability of individuals aged 60 and above declines by approximately 16% every decade, thereby heightening their vulnerability to falls (8). Furthermore, impaired balance and gait function serve as potent predictors of fall risk in this population, underscoring their significance in assessing an individual’s predisposition to falls (9). Consequently, by assessing gait and balance abilities in older adults, it is feasible to anticipate fall risk and subsequently implement targeted preventive measures (10).

Dance, a harmonious blend of rhythmic motions, embodies intricate emotional, physical, cognitive, and social dimensions (11). Its diverse styles, intricate steps, and accompanying melodies hold a particular appeal for older adults, motivating them to engage in extended practice sessions (12, 13). This practice has been shown to exert a salutary influence on various risk factors associated with falls, including enhancements in balance, gait dynamics, strength, and flexibility (14–16). This benefit is particularly pronounced among individuals with chronic health conditions (17–21).

While several meta-analyses have unequivocally affirmed dance’s efficacy in mitigating the risk of falls among the older adult (22, 23), a notable gap remains in the literature regarding a direct comparison of the improvement effects elicited by different dance forms. Thus, there is a pressing need to explore and compare the specific contributions of various dance styles to the overall wellbeing and fall prevention of older adults.

Network Meta-analysis, an evolution from the conventional Meta-analysis framework, offers a robust methodology for identifying the “optimal” intervention options by comprehensively comparing their direct and indirect effects (24). The present study employs Network Meta-analysis to evaluate the efficacy of various dance interventions in mitigating falls among older adults. Our objective is to provide evidence-based recommendations for effective dance-based interventions that can contribute to reducing fall risk in this population. By doing so, we aim to contribute to the development of tailored and targeted fall prevention strategies tailored specifically for older individuals.

## Methods

### Protocol and registration

This research followed the Preferred Reporting Items for Systematic Reviews and Meta-analyses (PRISMA) statement checklist (25, 26) and was registered in the International Prospective Register of Systematic Reviews (PROSPERO; CRD42022323426).

### Search strategy

The search was carried out in 8 November 2024 using the electronic databases CNKI, WanFang Data, PubMed, EBSCO, Web of Science and Cochrane Library. The search comprised the following terms and MeSH terms: dancing, fall, aged, older adult, randomized controlled trial, randomized, and placebo. No restrictions on language or publication type were applied. The electronic search was complemented by hand-searching the references of included papers and previous reviews.

### Eligibility criteria

This study included reports of published, peer-reviewed RCTS that investigated the effects of dance on balance and fall outcomes in older adults.

Types of participants: This study included only randomized controlled trials (RCTs) involving older adults aged 60 years and above. The health status of the participants was clearly reported in all included studies. The experimental arm of these trials featured a single dance intervention as the sole additional treatment compared to the control group. We excluded studies on specific conditions (e.g., hospitalized individuals), where the effects of the interventions cannot be generalized to most community-dwelling older adults.

Types of outcomes: (1) Berg Balance Scale (BBS): A validated tool assessing balance function and predictive of fall risk, with a total score of 56. Higher scores signify superior balance capabilities (27); (2) Timed Up and Go Test (TUG): Evaluates functional walking ability and fall risk by measuring the time taken for an individual to rise from a seated position, walk a distance of 3 meters, turn around, walk back, and sit down again. Shorter completion times indicate greater mobility (28). (3) Tinetti Scale (Tinetti): Comprehensively assesses mobility, balance, and gait, with a focus on predicting falls. It comprises both a balance test and a gait test, where higher scores reflect better balance and walking abilities (29). (4) Single-Leg Standing Test (SLS): A test of balance where participants, with hands either hanging naturally by their sides or placed flat to the left and right, stand on one foot with their eyes closed. The duration of maintaining this position is recorded, with shorter durations indicative of poorer balance (30).

Exclusion Criteria: (1) Academic dissertation, conference thesis and thesis abstract; (2) Literature where the mean and standard deviation of outcome indicators cannot be calculated; (3) Non-compliance with outcome indicators; (4) Full text is not available and failed to contact the author.

### Data extraction

The data extraction process for the included studies was conducted independently by two researchers, with a focus on the following variables: (1) Study characteristics, encompassing the author, year of publication, and study design, as well as the sample demographics including size, gender distribution, and age range; (2) A detailed description of the intervention program, specifically the type of dance intervention, its frequency, and duration, along with the characteristics of the control group; (3) Pertinent outcome data, including the mean values and standard deviations of the primary outcome indicators. Any discrepancies encountered during the extraction process were resolved through consensus or, if necessary, by consulting a third reviewer.

### Risk of bias within studies

The risk of bias in all included studies was independently evaluated by two reviewers utilizing the Cochrane Collaboration’s risk of bias tool. Any discrepancies encountered during this process were resolved through thorough discussion and negotiation. In cases where consensus could not be reached, a third investigator was consulted for assistance in adjudicating the risk assessment (25).

The literature evaluation encompassed five key aspects: randomization method, allocation concealment, blinding of participants and/or personnel, blinding of outcome assessment, and selection of reported results. Studies that explicitly described these characteristics in their published documents were deemed to have met the criteria and were subsequently classified as either “low risk” or “high risk” of bias. Conversely, studies that failed to provide sufficient information on these aspects were classified as having an “unclear risk” of bias.

### Statistical analysis

Network meta-analysis was performed using a frequentist framework, implemented in the Stata 17.0 software package. For continuous outcomes, the mean difference (MD) with 95% confidence intervals (CIs) was employed as the measure of effect. Studies of older adults with different health status were analyzed separately.

To visually depict the comparative relationships among dancing interventions and control groups, a network diagram was constructed, wherein the thickness of the connecting lines represented the number of comparative studies between any two interventions. The surface under the cumulative ranking curve (SUCRA) served as the ranking index, with 100% indicating the most efficacious intervention and 0% suggesting the least effective or ineffectual intervention (31).

Furthermore, funnel plots were generated to scrutinize potential small-study effects across the included studies, ensuring a rigorous assessment of the data and enhancing the credibility of our findings.

## Results

### Search results

**Figure 1:**
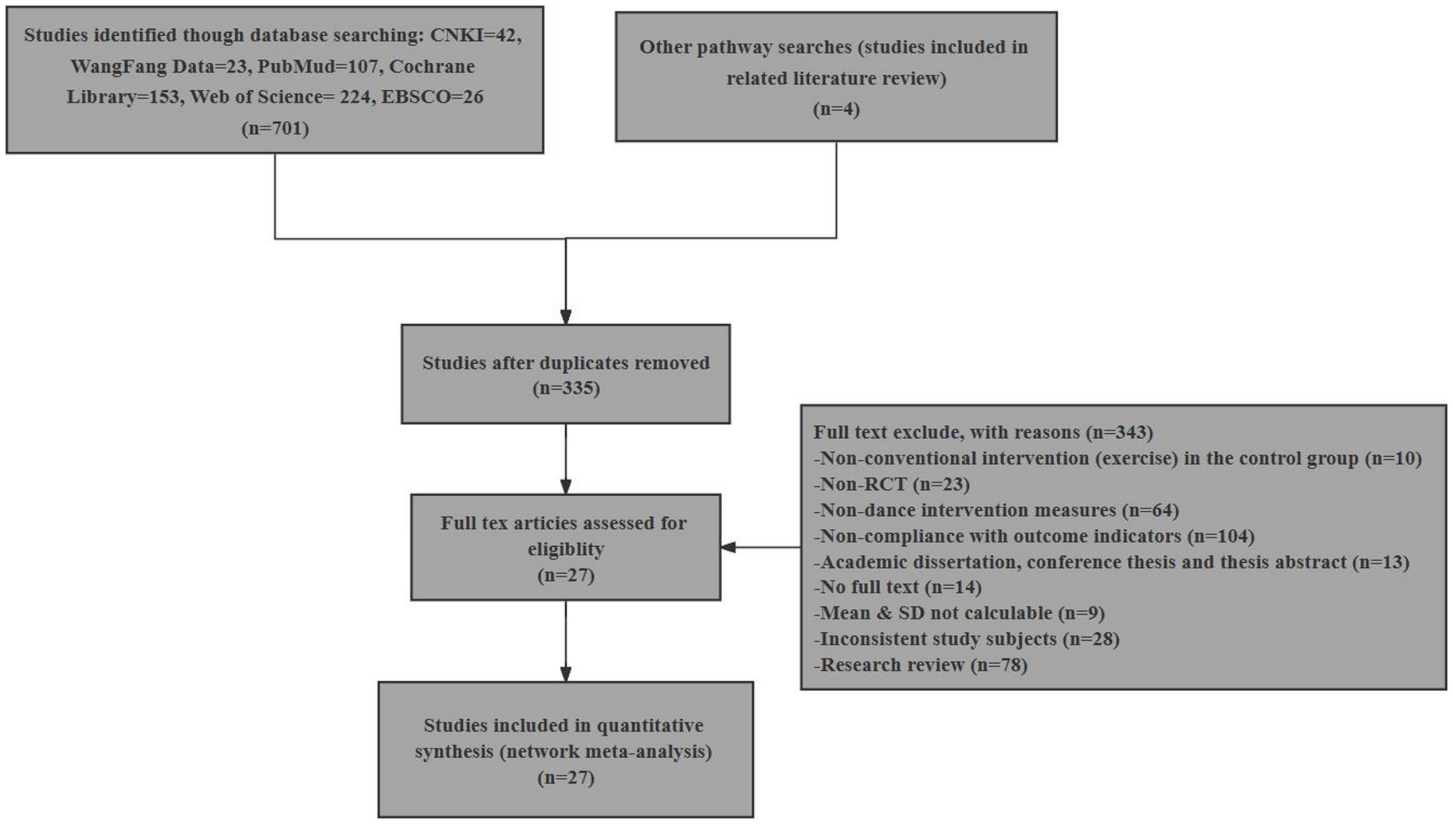
Study selection flowchart according to the Preferred Reporting Items for Systematic Reviews and Meta-Analyses (PRISMA) statements.

The initial search process yielded a comprehensive pool of 701 articles, comprising 42 articles retrieved from CNKI, 23 from WanFang Data, 107 sourced from PubMed, 153 from the Cochrane Library, 224 from Web of Science, 26 from EBSCO, and an additional 4 articles discovered through alternative search pathways (specifically, studies incorporated in relevant literature reviews). Following a rigorous and meticulous layer-by-layer screening procedure, a final selection of 27 articles (11, 32–57) was deemed to fulfill the stringent inclusion criteria established for this review (Figure 1).

### Characteristics of the included studies

The characteristics of the included studies are presented in Table 1. Among the studies included in this review. The study population encompassed 1,219 participants. Twelve of which the studies included healthy older adults (such as older adults who live independently in the community; n = 548). Eleven studies included patients with Parkinson’s disease (n = 341). One study included people with Mild Cognitive Impairment (n = 32). One study included people with chronic cardiac failure (n = 57). Two studies included long-term nursing home residents, which was classified as function decline (n = 241).

**Table 1:** Basic characteristics of the included studies.

Study	Subject status	Group	*N*	Gender (male/female)	Mean age (years)	Duration (weeks)	Frequency (days/week)	Class duration (min)	Outcome index
Shigematsu et al. (32)	Healthy	Aerobic dance	20	0/20	78.6 ± 4.0	12	3	60	SLS
		Regular activities	18	0/18	79.8 ± 5.0				
Federici et al. (33)	Healthy	Caribbean dance	20	N/A	62.7 ± 4.1	12	2	60	Tinetti
		Regular activities	20	N/A	63.5 ± 3.7				
Eyigor et al. (34)	Healthy	Turkish folk dance	19	0/19	73.5 ± 7.6	8	3	60	BBS
		Regular activities	18	0/18	71.2 ± 5.5				
Hackney & Earhart (35)	Parkinsonism	Ballroom dance (Tango)	17	11/6	68.2 ± 1.4	13	2	60	BBS, TGU
		Regular activities	17	12/5	66.5 ± 2.8				
Hackney & Earhart (35)	Parkinsonism	Ballroom dance (Waltz and foxtrot)	17	11/6	68.2 ± 1.4	13	2	60	BBS, TGU
		Regular activities	17	12/5	66.5 ± 2.8				
Holmerová et al. (36)	Functional Decline	Ballroom dance	27	2/25	81.0 ± 9.6	12	1	75	TUG
		Regular activities	25	4/21	82.8 ± 7.5				
Keogh et al. (37)	Healthy	Modern dance	18	1/17	69.1 ± 6.5	12	1	N/A	TUG
		Regular activities	13	1/12	71.5 ± 7.4				
Keogh et al. (37)	Healthy	Modern dance	18	1/17	69.1 ± 6.5	12	2	N/A	TUG
		Regular activities	13	1/12	71.5 ± 7.4				
McKee et al. (38)	Parkinsonism	Ballroom dance	24	12/12	68.4 ± 7.5	12	2	90	BBS
		Health education	9	8/1	74.4 ± 6.5				
Volpe et al. (39)	Parkinsonism	Irish dance	12	7/5	61.6 ± 4.5	24	1	90	BBS
		Usual care	12	6/6	64.0 ± 5.3				
Kaltsatou et al. (40)	Chronic heart failure	Greek traditional dance	18	18/0	67.2 ± 4.2	32	3	60	BBS
		Regular activities	16	16/0	67.5 ± 5.0				
Cepeda et al. (41)	Healthy	Ballroom dance	19	0/19	69.1 ± 6.5	8	3	60	TUG
		Regular activities	15	0/15	71.5 ± 7.4				
Cruz-Ferreira et al. (42)	Healthy	Creative dance	32	0/32	71.1 ± 3.9	24	3	50	TUG
		Regular activities	25	0/25	72.8 ± 4.5				
Hashimoto et al. (43)	Parkinsonism	Ballroom dance	15	3/12	67.9 ± 7.0	12	1	60	BBS, TUG
		Regular activities	14	7/7	69.7 ± 4.0				
Machacova et al. (44)	Functional Decline	Ballroom dance	92	6/86	83.03 ± 9.10	12	1	60	TUG
		Regular activities	97	11/86	82.88 ± 8.16				
Romenets et al. (45)	Parkinsonism	Ballroom dance (Tango)	18	12/6	63.2 ± 9.9	12	2	60	TUG
		Usual care	15	7/8	64.3 ± 8.1				
Serrano-Guzmán et al. (46)	Postmenopausal	Spanish folk dance	27	0/27	69.1 ± 4.4	8	3	50	TUG
		Health education	25	0/25	69.5 ± 3.2				
Ventura et al. (47)	Parkinsonism	Dance therapy (DfPD)	8	0/8	71, 8 ± 3.6	50	1	85	TUG
		Usual care	7	2/5	70.4 ± 5.5				
Kunkel et al. (48)	Parkinsonism	Ballroom dance	36	19/17	71.3 ± 7.7	10	2	60	BBS, TUG
		Usual care	15	9/6	69.7 ± 6.0				
Bennett & Hackney (49)	Sedentary and had no disability	Line dance	12	2/10	65.0 ~ 93.0	8	2	60	BBS
		Usual care	11	1/10	65.0 ~ 93.0				
Lee et al. (50)	Parkinsonism	Qi dance	25	10/15	65.8 ± 7.2	8	2	60	BBS
		Usual care	16	7/9	65.7 ± 6.4				
Leelapattana et al. (51)	Healthy	Thai classical dance	19	0/19	66.4 ± 4.2	12	7	10	TUG
		Swing arm exercise	20	0/20	66.9 ± 5.6				
Michels et al. (52)	Parkinsonism	Dance Therapy	9	6/7	66.4	10	1	69	BBS, TUG
		Health education	3		75.5				
Joung & Lee (53)	Healthy	Creative dance	41	N/A	70.5 ± 7.9	8	2	90	BBS, TUG
		Stretching exercise	41	N/A	71.8 ± 7.8				
Qi et al. (54)	Mild cognitive impairment	Mild cognitive impairment	16	5/11	70.6 ± 6.2	12	3	35	BBS
		Usual care	16	4/12	69.1 ± 8.1				
Solla et al. (55)	Parkinsonism	Sardinian folk dance	10	6/4	67.8 ± 5.9	12	2	90	BBS, TUG
		Usual care	10	7/3	67.1 ± 6.3				
Franco et al. (11)	Healthy	Aerobic dance	35	1/34	68.6 ± 7.2	12	2	60	SLS
		Health education	36	5/31	70.0 ± 6.2				
Kalyani et al. (56)	Parkinsonism	Dance therapy (DfPD)	17	3/14	65.24 ± 11.88	12	2	60	BBS,TUG
		Usual care	16	10/6	66.50 ± 7.70				
Rodziewicz-Flis et al. (57)	Healthy	Polish folk dance	10	0/10	72.1 ± 4.1	12	3	50	TUG
		Regular activities	10	0/10	73.4 ± 5.0				

N/A: not available.

The types of dance performed in the studies were ballroom dancing (i.e., Tango, Waltz and Fox trot), aerobic dance (i.e., Line dance and Senior Dance), Creative dance, Folk dance (i.e., Caribbean Dance, Turkish Folk Dance, Spanish folk dance, Polish folk dance, Irish dancing, Sardinian folk dance, Classical dance and Traditional Greek dance), and Dance therapy.

The intervention duration spanned from 8 to 50 weeks, with individual sessions lasting between 10 and 100 min. The frequency of interventions varied, ranging from once to seven times per week (Table 1).

### Assessment of methodological quality

**Figure 2:**
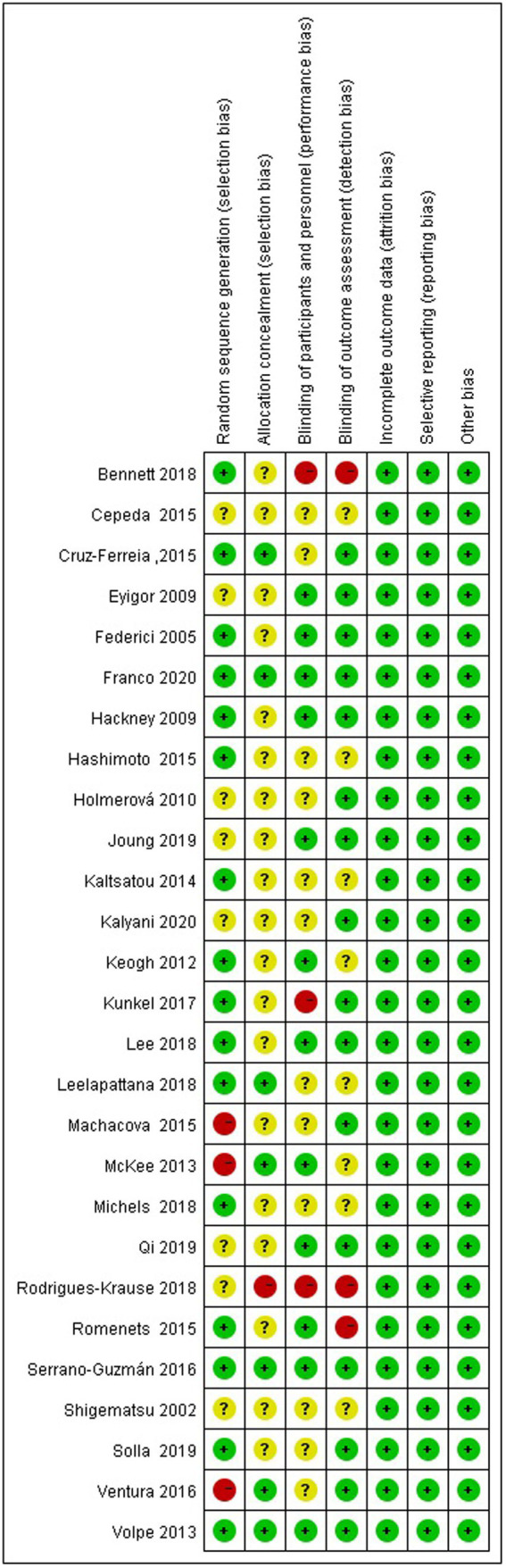
Risk of bias summary.

The risk-of-bias assessment for the 16 studies that explicitly reported randomized methods revealed that seven of these studies also documented allocation concealment measures, while 12 studies reported blinding protocols for participants, personnel involved in the study, and outcome assessors (Figure 2; Table 2).

**Table 2:** Cochrane risk of bias evaluation results.

Studies	Randomization process	Allocation hiding	Blinding to participants and staff	Blinding to the outcome assessors	Integrity of the resulting data	Selectivity report	Other sources of bias
Shigematsu et al. (32)	Unclear	Unclear	Unclear	Unclear	Low	Low	Low
Federici et al. (33)	Low	Unclear	Low	Low	Low	Low	Low
Eyigor et al. (34)	Unclear	Unclear	Low	Low	Low	Low	Low
Hackney & Earhart (35)	Low	Unclear	Low	Low	Low	Low	Low
Holmerová et al. (36)	Unclear	Unclear	Unclear	Low	Low	Low	Low
Keogh et al. (37)	Low	Unclear	Low	Unclear	Low	Low	Low
McKee et al. (38)	High	Low	Low	Unclear	Low	Low	Low
Volpe et al. (39)	Low	Low	Low	Low	Low	Low	Low
Kaltsatou et al. (40)	Low	Unclear	Unclear	Unclear	Low	Low	Low
Cepeda et al. (41)	Unclear	Unclear	Unclear	Unclear	Low	Low	Low
Cruz-Ferreia et al. (42)	Low	Low	Unclear	Low	Low	Low	Low
Hashimoto et al. (43)	Low	Unclear	Unclear	Unclear	Low	Low	Low
Machacova et al. (44)	High	Unclear	Unclear	Low	Low	Low	Low
Romenets et al. (45)	Low	Unclear	Low	High	Low	Low	Low
Serrano-Guzmán et al. (46)	Low	Low	Low	Low	Low	Low	Low
Ventura et al. (47)	High	Low	Unclear	Low	Low	Low	Low
Kunkel et al. (48)	Low	Unclear	High	Low	Low	Low	Low
Bennett & Hackney (49)`	Low	Unclear	High	High	Low	Low	Low
Lee et al. (50)	Low	Unclear	Low	Low	Low	Low	Low
Leelapattana et al. (51)	Low	Low	Unclear	Unclear	Low	Low	Low
Michels et al. (52)	Low	Unclear	Unclear	Unclear	Low	Low	Low
Joung & Lee (53)	Unclear	Unclear	Low	Low	Low	Low	Low
Qi et al. (54)	Unclear	Unclear	Low	Low	Low	Low	Low
Solla et al. (55)	Low	Unclear	Unclear	Low	Low	Low	Low
Franco et al. (11)	Low	Low	Low	Low	Low	Low	Low
Kalyani et al. (56)	Unclear	Unclear	Unclear	Low	Low	Low	Low
Rodziewicz-Flis et al. (57)	Unclear	Unclear	Unclear	Unclear	Low	Low	Low

### Network meta-analysis

One study on chronic heart failure populations (40), one study on mild cognitive impairment (54), and two studies on nursing home residents (36, 44) were excluded from the network meta-analysis. The reasons for this exclusion were the heterogeneous health status of the study subjects and the fact that the premise of network meta-analysis necessitates comparisons among three or more interventions.

**Figure 3:**
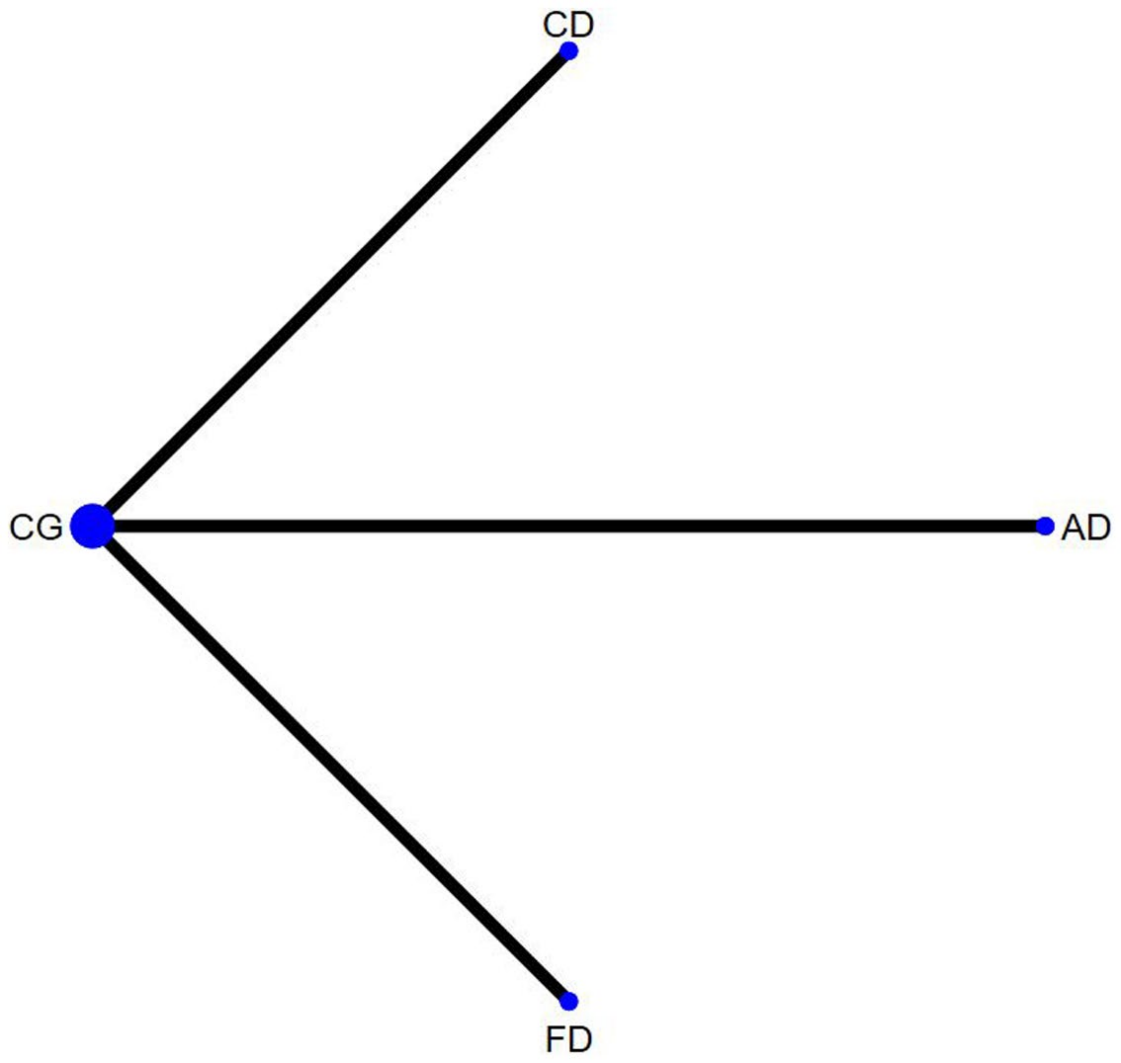
Berg balance scale network diagram for healthy older adults.

**Figure 4:**
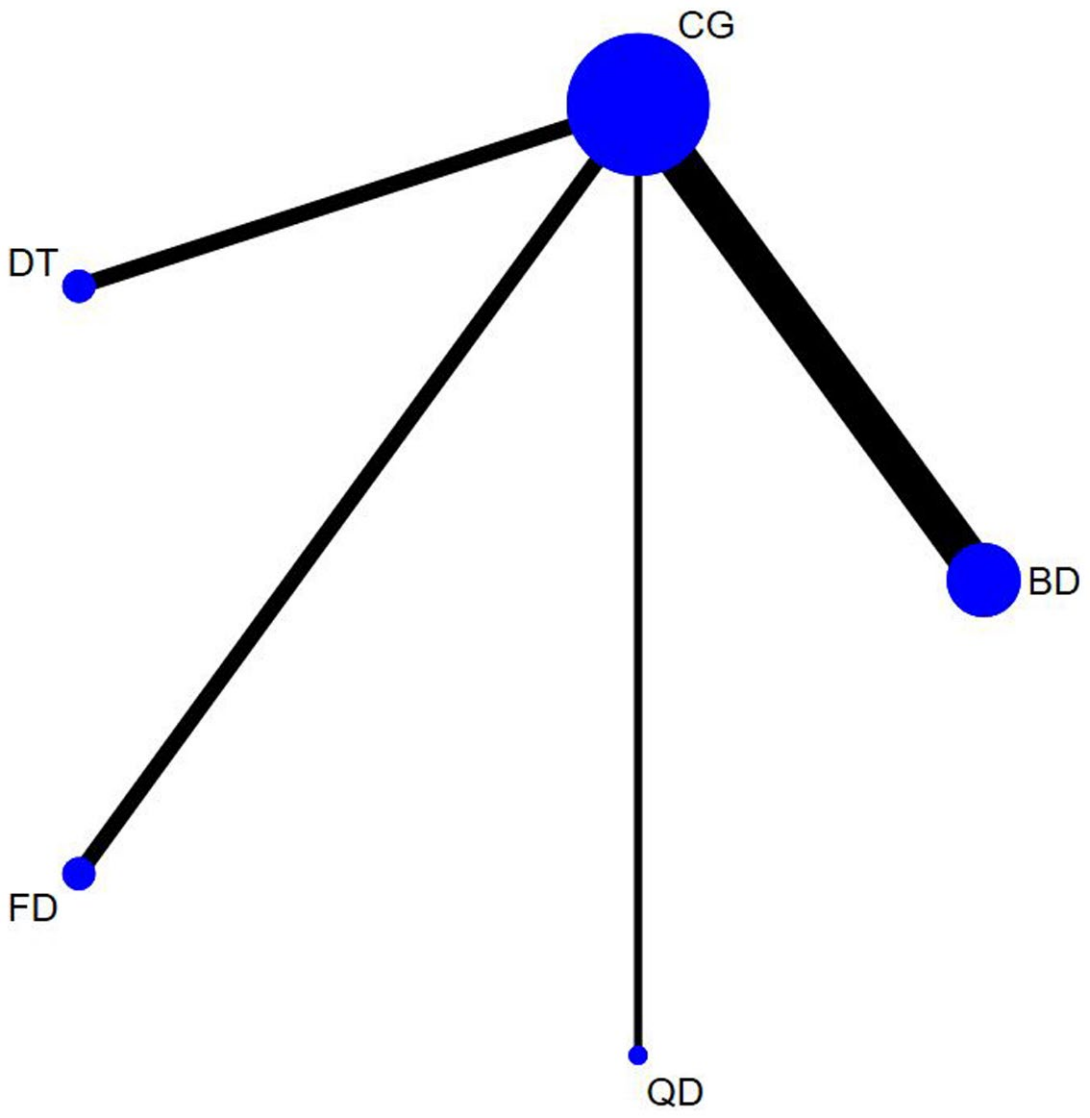
Timed up and go network diagram for patients with parkinson’s disease.

The outcome measures encompassed 12 studies (13 comparisons) (34, 35, 38, 39, 43, 48–50, 52, 53, 55, 56) that assessed BBS scores. In a healthy population, three studies (34, 49, 53) with three interventions were included: Creative dance (CD), Aerobic dance (AD) and Folk dance (FD; Figure 3). In patients with Parkinson’s disease, there were nine studies (10 comparisons) (35, 38, 39, 43, 48, 50, 52, 55, 57) with a total of four interventions: Dance Therapy (DT), FD, Qi dance (QD) and Ballroom dance (BD), of which BD had the thickest line in the visualization with the control group, indicating that it was the most frequently studied intervention in this context (Figure 4).

**Figure 5:**
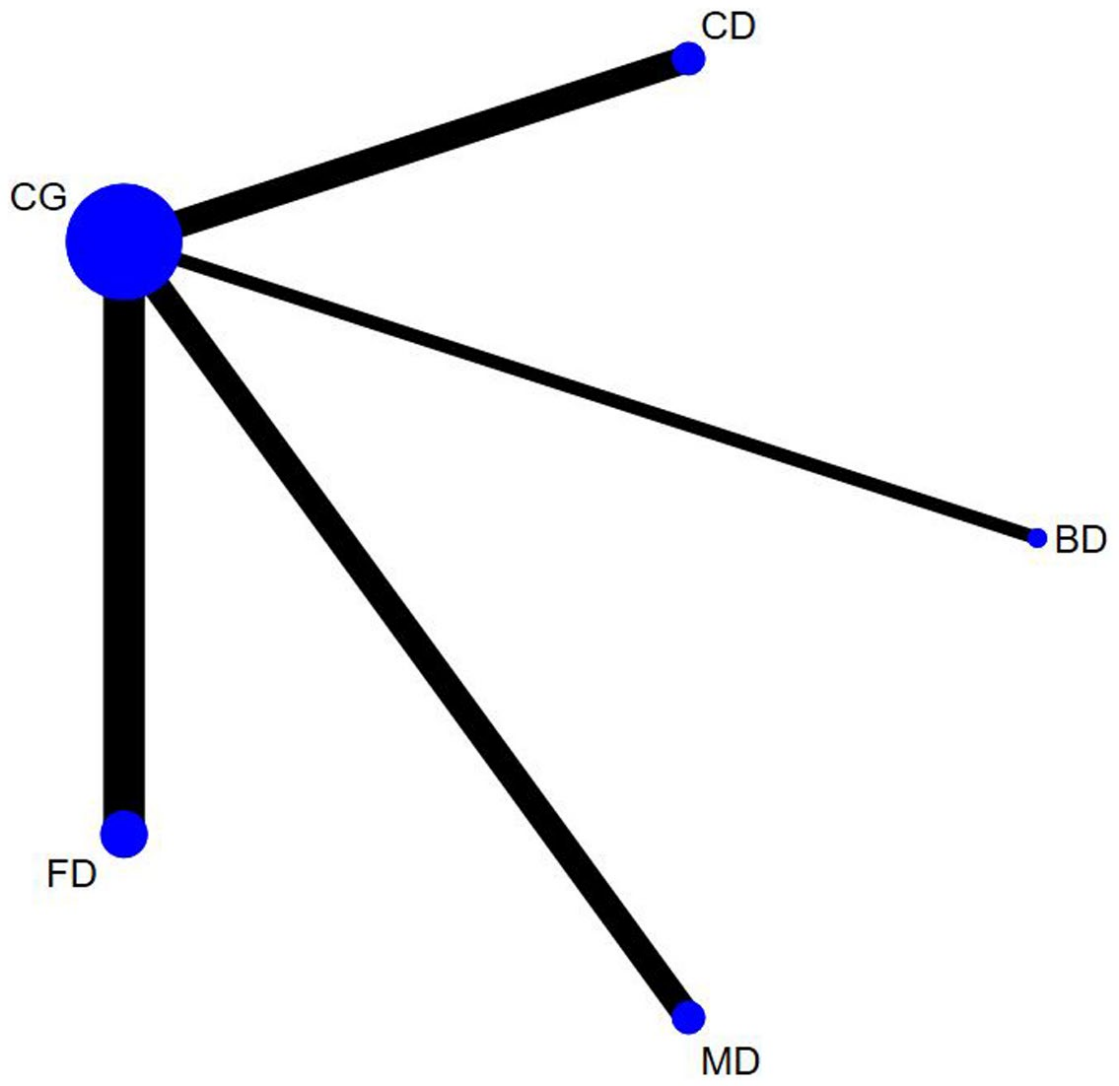
Timed up and go test network diagram for healthy older adults.

**Figure 6:**
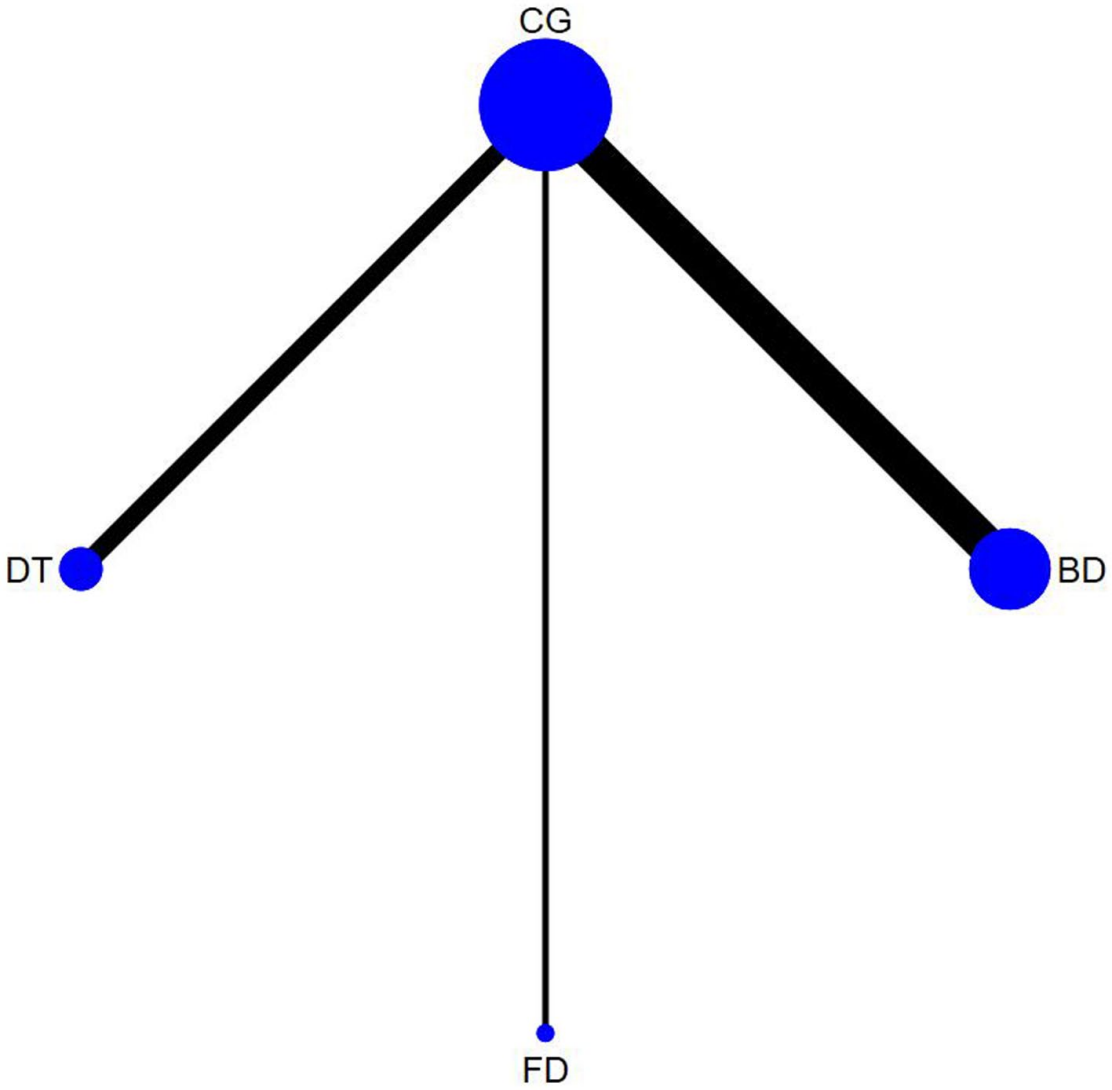
Berg balance scale network diagram for patients with parkinson’s disease.

The outcome measures encompassed 16 studies (18 comparisons) (35, 37, 38, 41–43, 45–48, 50, 52, 53, 55–57) that assessed TUG scores. In a healthy population, seven studies (8 comparisons) (37, 41, 42, 46, 50, 53, 57) with four interventions were included: FD, Modern dance (MD), BD, and CD. Of these FD had the thickest line in the visualization with the control group, indicating that it was the most frequently studied intervention in this context (Figure 5). In patients with Parkinson’s disease, there were nine studies (10 comparisons) (35, 38, 43, 45, 47, 48, 52, 55, 56) with a total of three interventions: DT, FD, and BD. Similarly, Ballroom dance (BD) had the thickest line in the visualization with the control group, indicating that it was the most frequently studied intervention in this context (Figure 6).

Given that the SLS time (11, 32, 46) and Tinetti score (33) assessments encompassed only a single intervention, the application of Network Meta-analysis was deemed inappropriate and, therefore, not performed.

#### Network meta-analysis of berg balance scale

**Figure 7:**
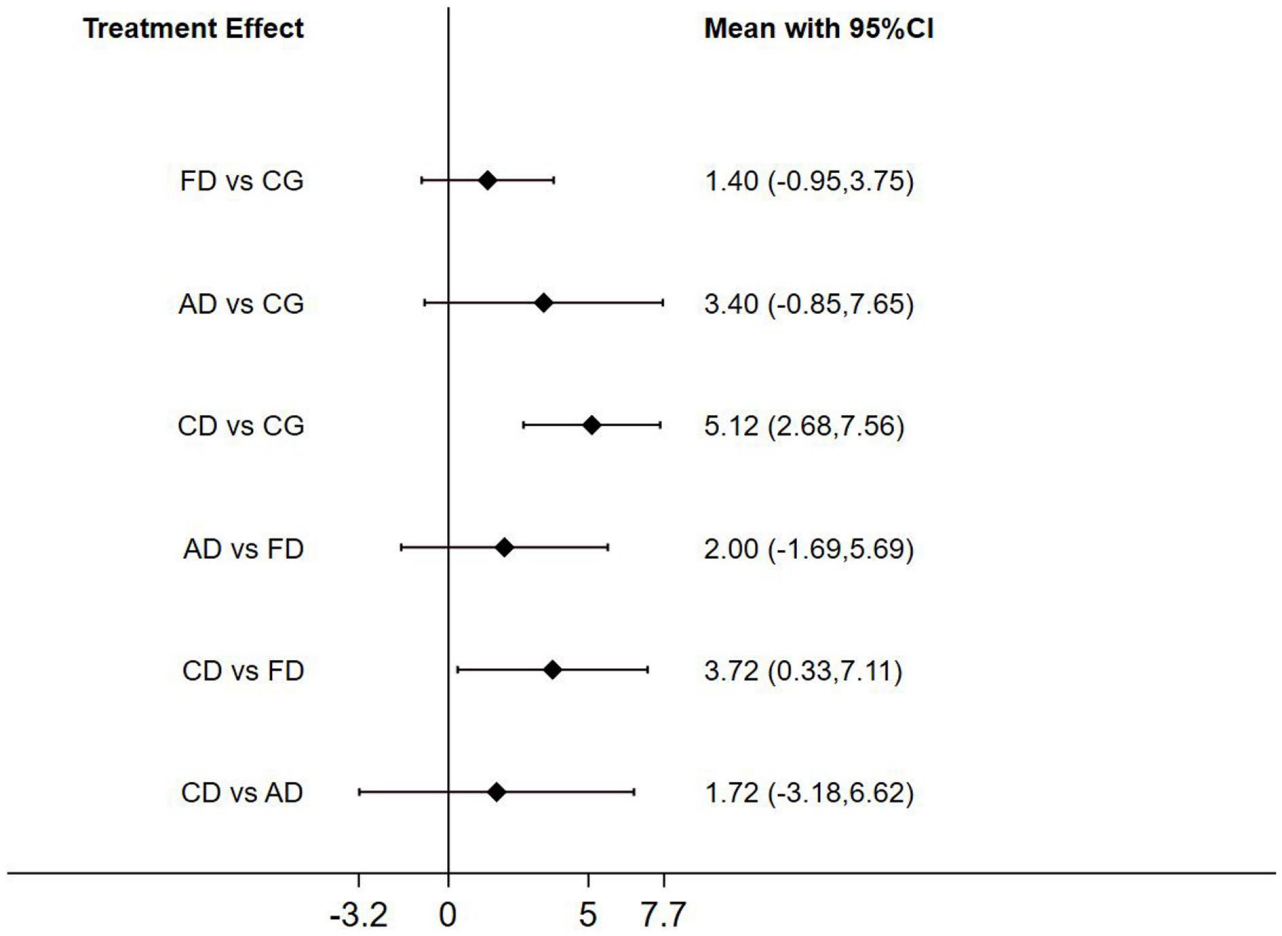
Forest plot of berg balance scale for healthy older adults.

The Network Meta-analysis (NMA) of Berg balance scale (BBS) scores revealed that Creative dance (CD) significantly outperformed the control group (CG) in health older adults (*p* < 0.05). In pairwise analyses comparing the three dance interventions, notable differences in intervention effects emerged between Folk dance and Creative dance (CD vs. FD, *p* < 0.05; Figure 7).

**Figure 8:**
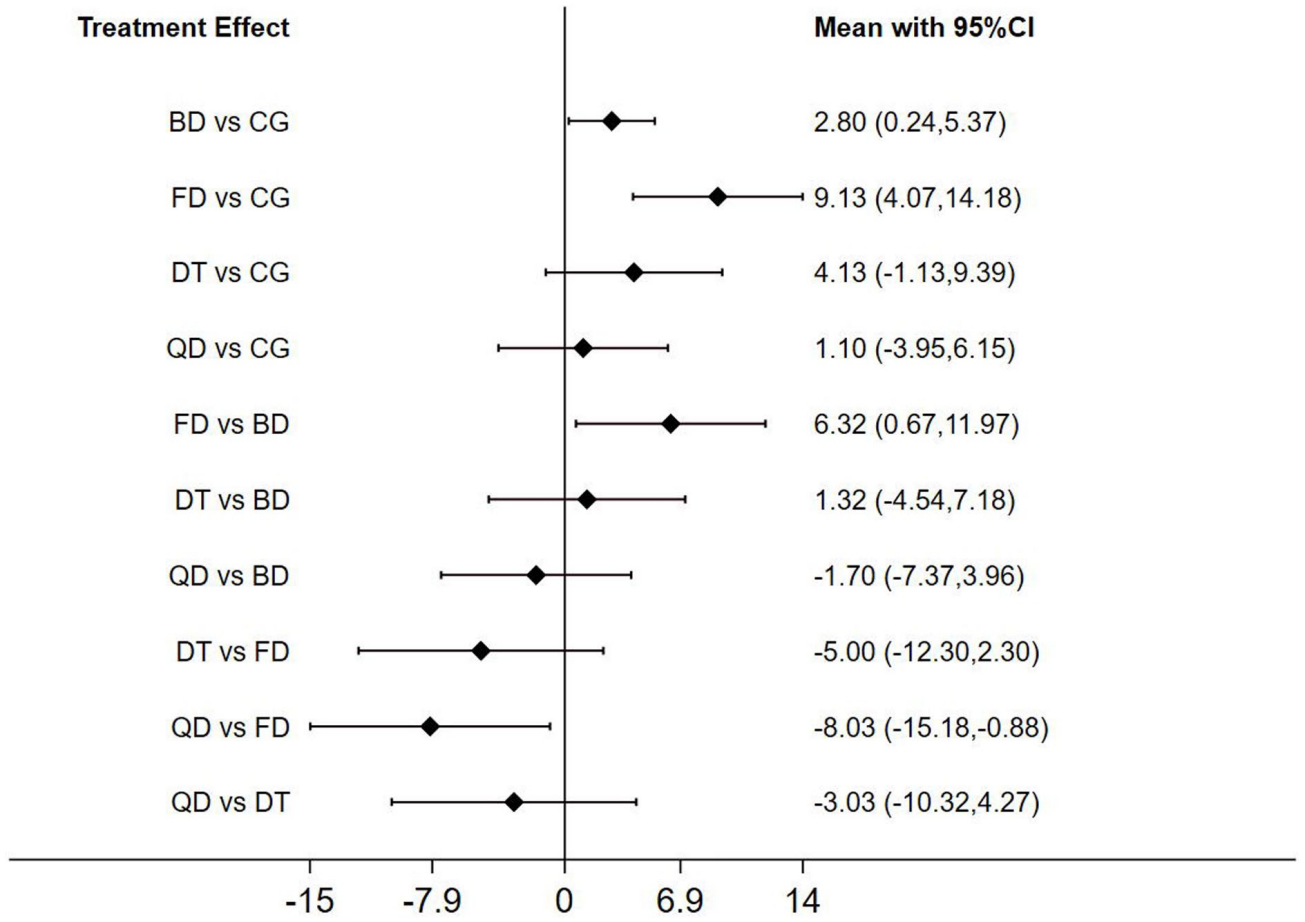
Forest plot of berg balance scale for patients with parkinson’s disease.

The NMA of BBS scores revealed that Ballroom dance (BD) and Folk dance (FD) significantly outperformed the control group (CG) in patients with Parkinson’s disease (*p* < 0.05). In pairwise analyses comparing the three dance interventions, notable differences in intervention effects emerged between Folk dance and Ballroom dance (FD vs. BD, *p* < 0.05), notable differences in intervention effects emerged between Qi dance and Folk dance (QD vs. FD, *p* < 0.05; Figure 8).

#### Network meta-analysis of timed up and go test

**Figure 9:**
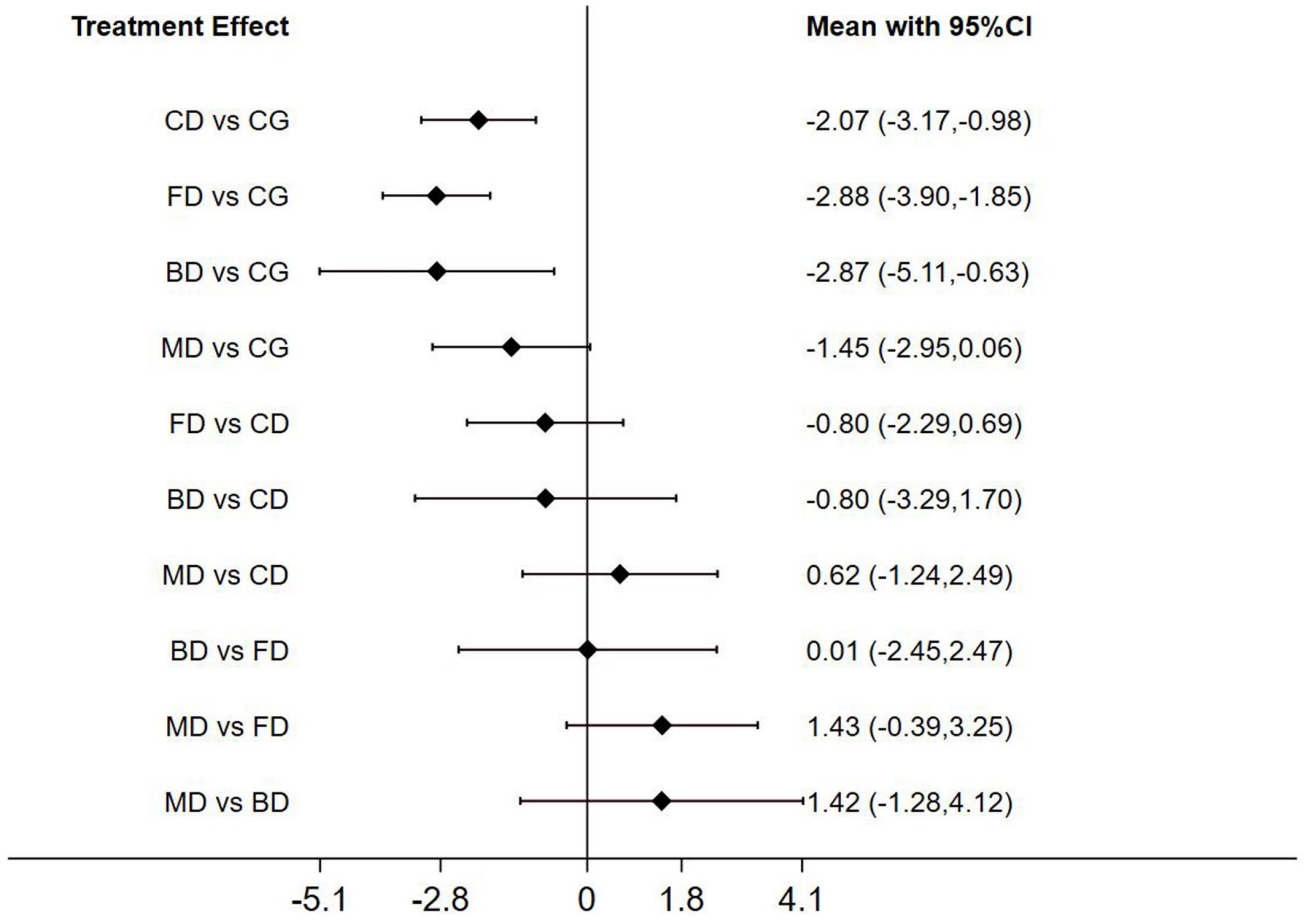
Forest plot of timed up and go for healthy older adults.

The Network Meta-analysis (NMA) of Timed Up Go (TUG) time revealed that Creative dance (CD; *p* < 0.01), Folk dance (FD; *p* < 0.01) and Ballroom dance (BD; *p* < 0.05) significantly outperformed the control group (CG) in health older adults (*p* < 0.01). In pairwise analyses comparing the three dance interventions, there was no significant difference in the intervention effect of each intervention (Figure 9).

**Figure 10:**
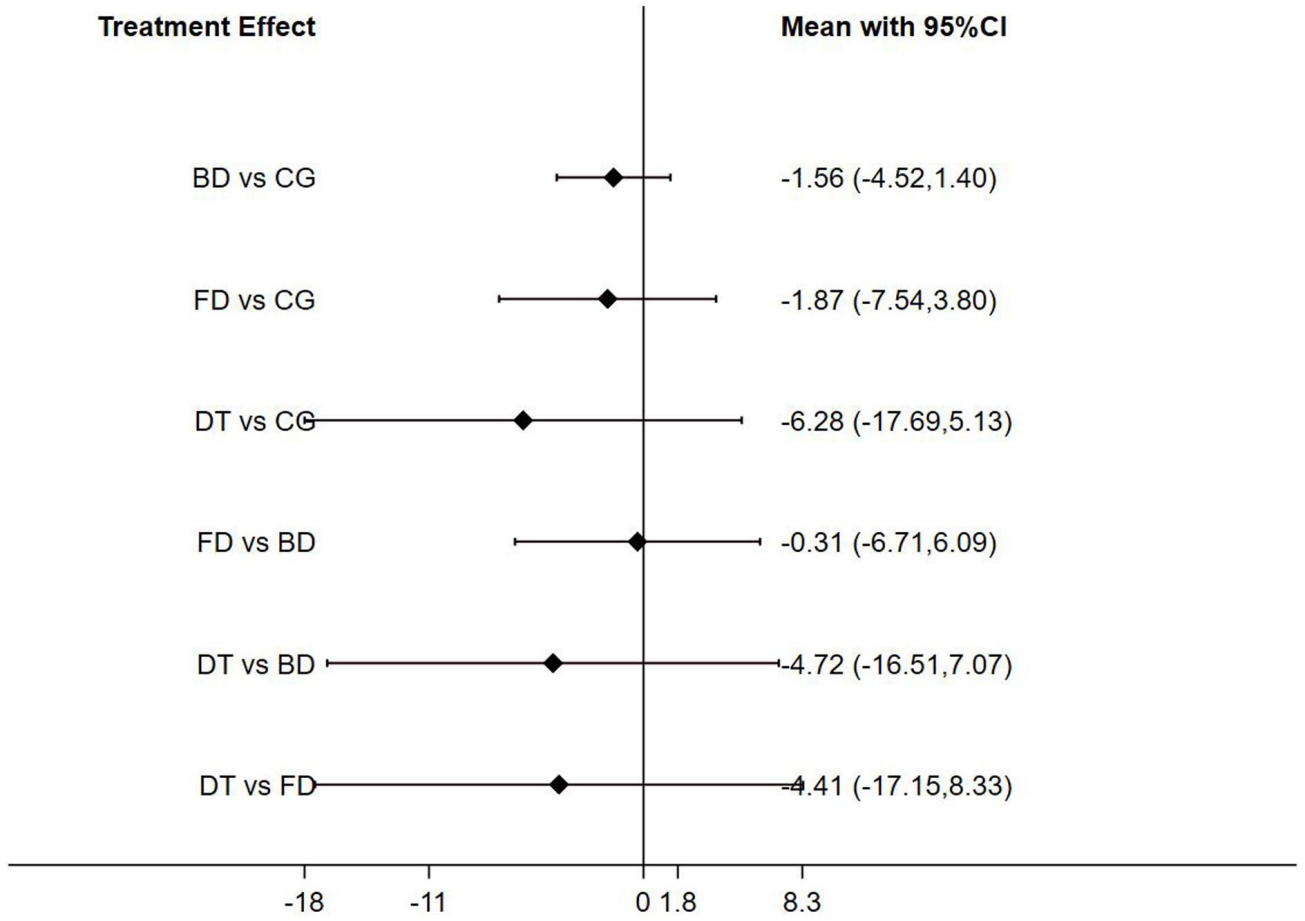
Forest plot of timed up and go for patients with parkinson’s disease.

The NMA results of TUG time showed that there were no significant differences between the interventions and the control group or between interventions in patients with Parkinson’s disease (*p* > 0.05; Figure 10).

### Ranking of mesh meta-analysis results for different interventions

**Figure 11:**
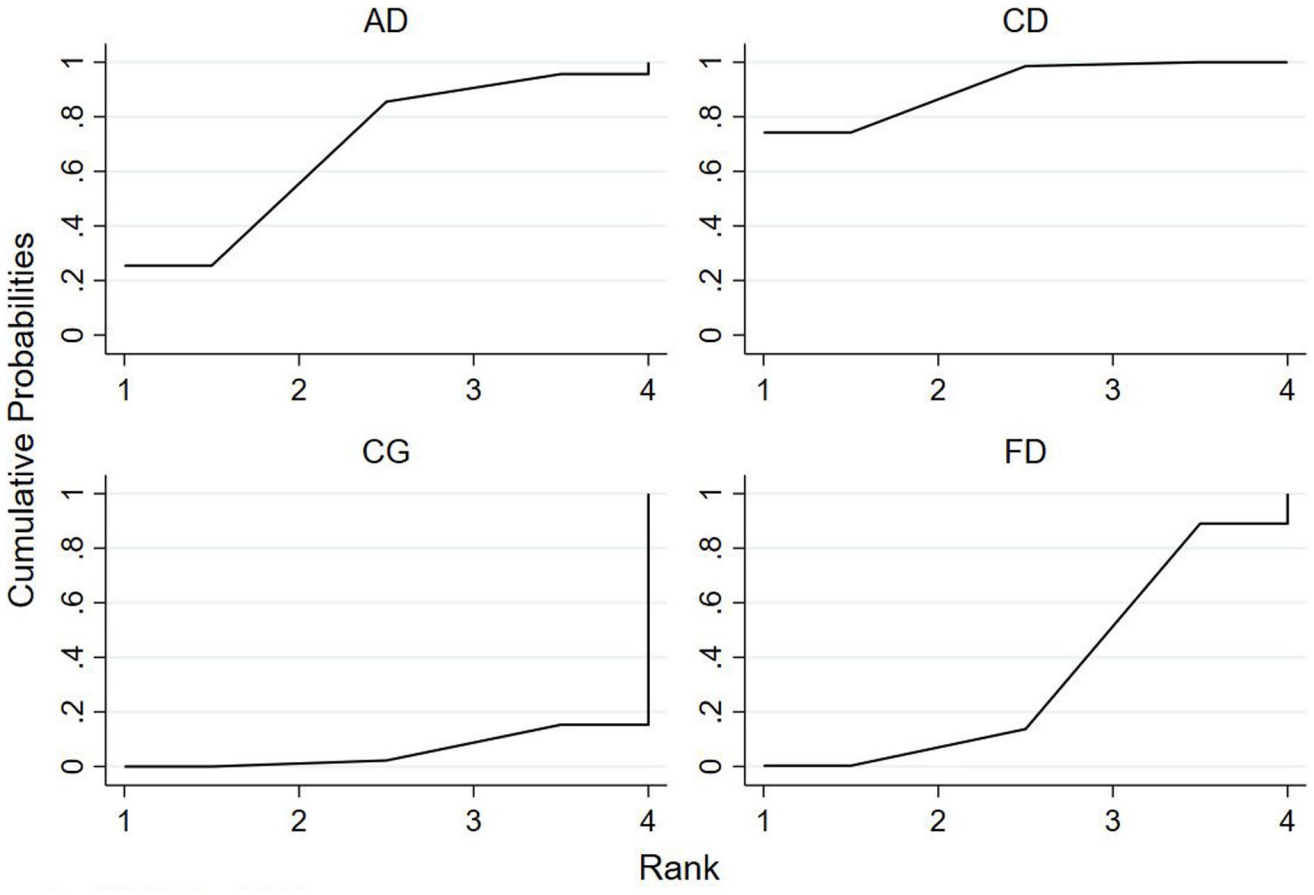
For healthy older adults, the surface under the cumulative ranking curve (SUCRA) in BBS.

**Figure 12:**
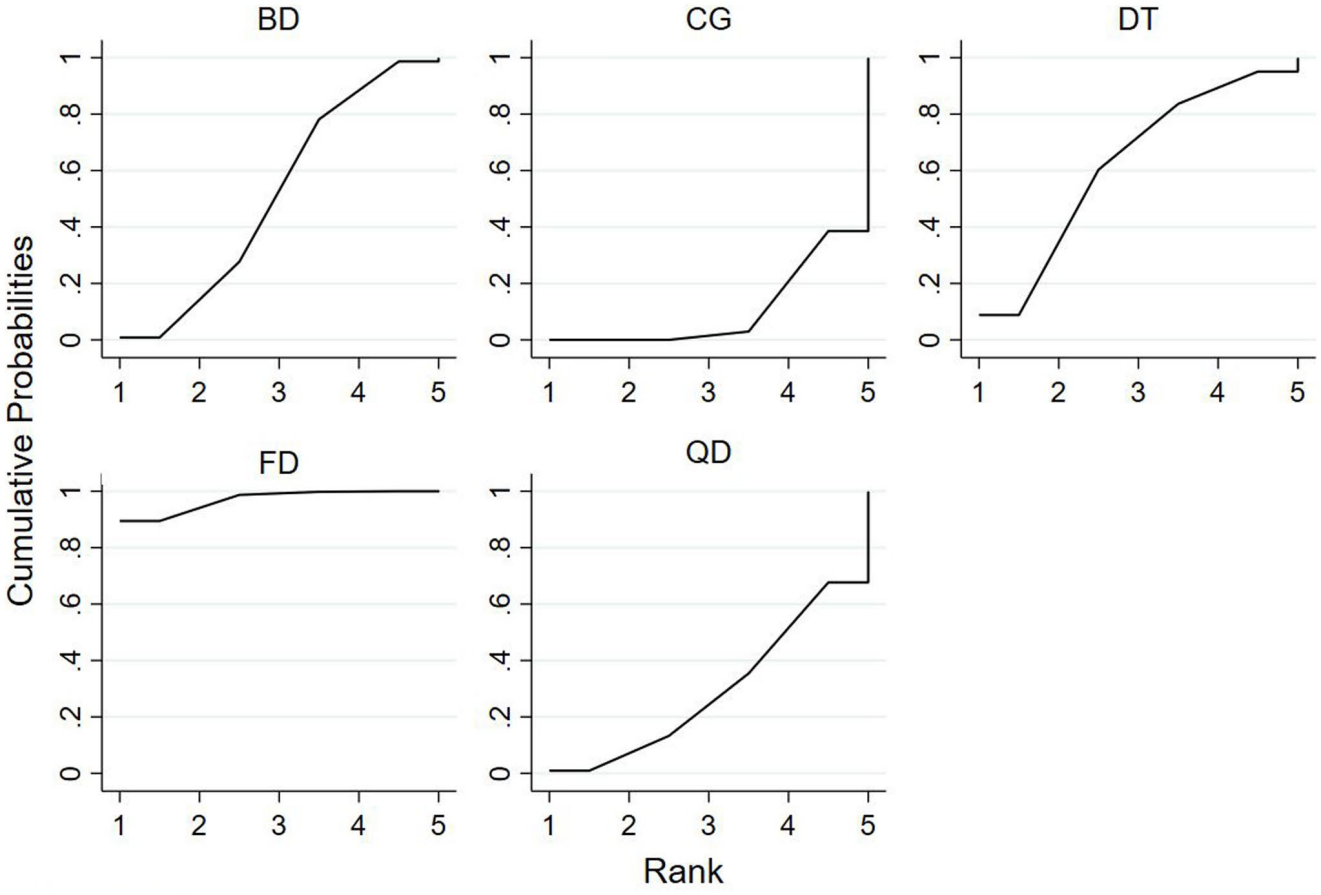
For patients with parkinson’s disease, the surface under the cumulative ranking curve (SUCRA) in BBS.

The relative rankings of the intervention measures with passive control as the reference. For the Berg Balance Scale (BBS) scores, Creative dance (90.9%) ranked best followed by Aerobic dance (68.9%) and Folk dance (34.3%) in health older adults (Figure 11). Combining the results with the forest plot, it is evident that the interventions of Creative dance significantly outperform Folk dance. Folk dance ranked best (97.1%) followed by Dance therapy (61.9%), Ballroom dance (51.4%) and Qi dance (29.0%) in patients with Parkinson’s disease. Combining the results with the forest plot, it is evident that the interventions of Folk dance significantly outperform Ballroom dance and Qi dance (Figure 12).

**Figure 13:**
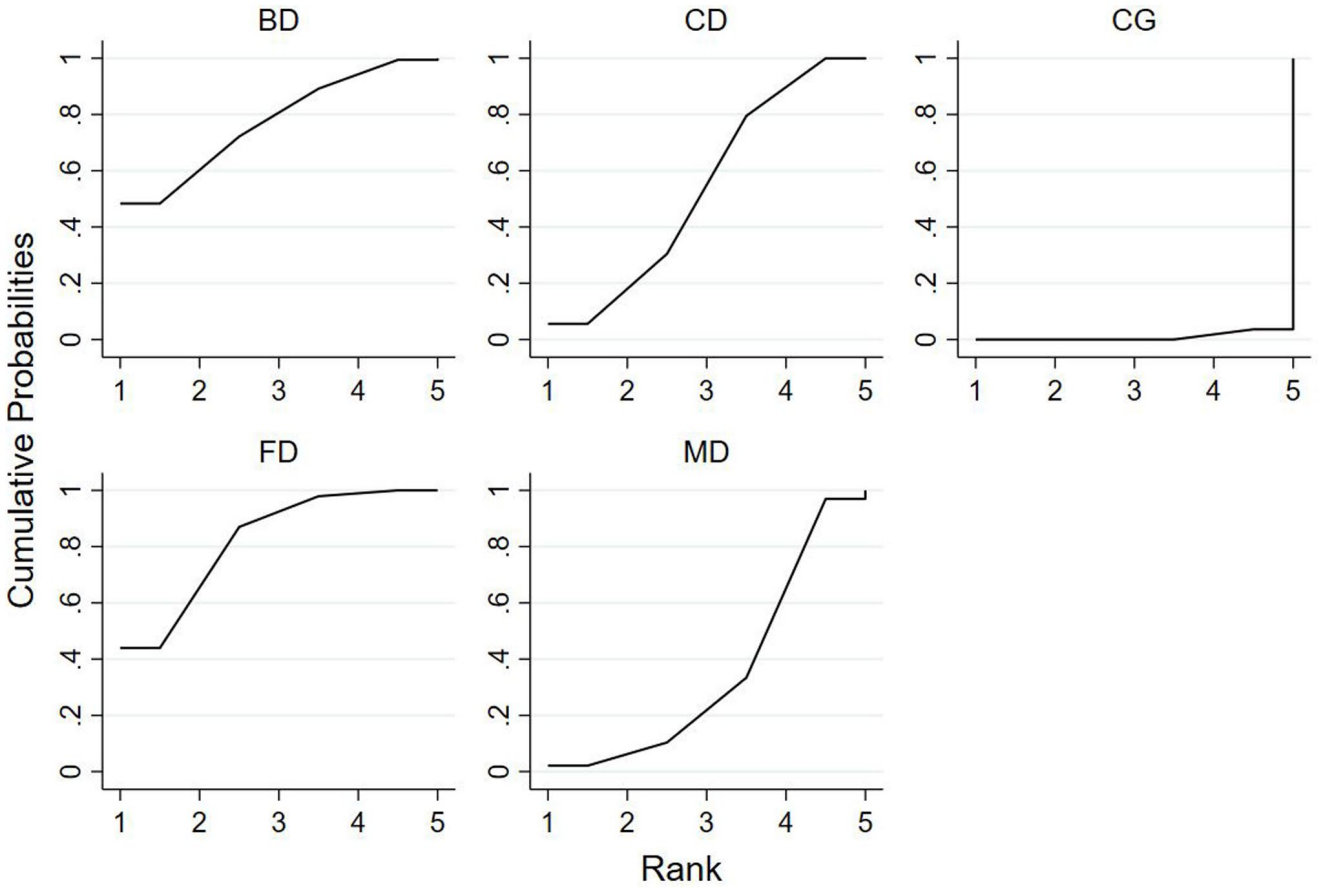
For healthy older adults, the surface under the cumulative ranking curve (SUCRA) in TUG.

**Figure 14:**
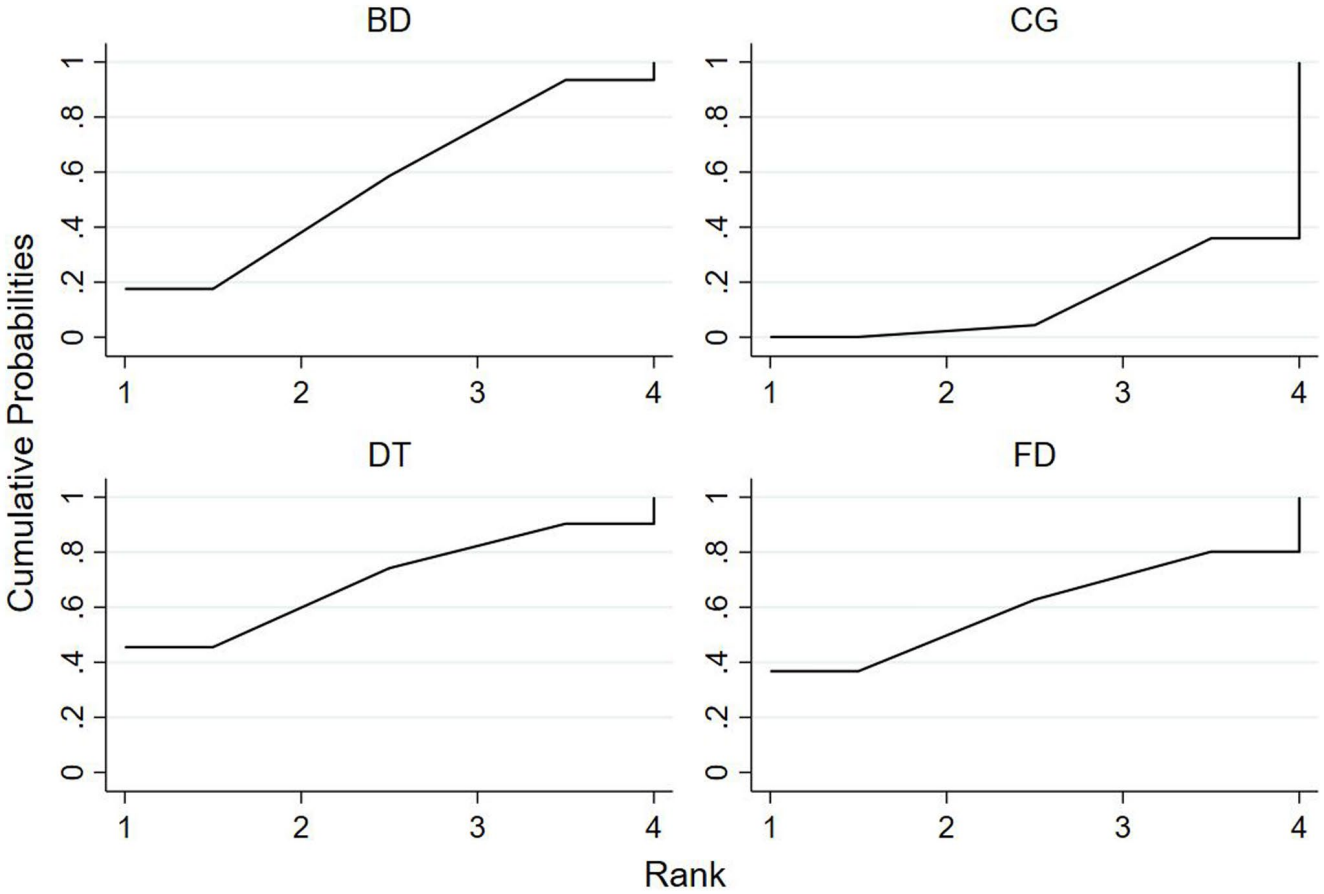
For patients with parkinson’s disease, the surface under the cumulative ranking curve (SUCRA) in TUG.

For TUG time, Folk dance (82.4%) ranked best followed by Ballroom dance (76.8%), Creative dance (54.1%) and Modern dance (35.8%) in health older adults (Figure 13). Dance therapy (70.0%) ranked best followed by Folk dance (59.9%) and Ballroom dance (56.6%) in patients with Parkinson’s disease (Figure 14).

### Publication bias analysis

**Figure 15:**
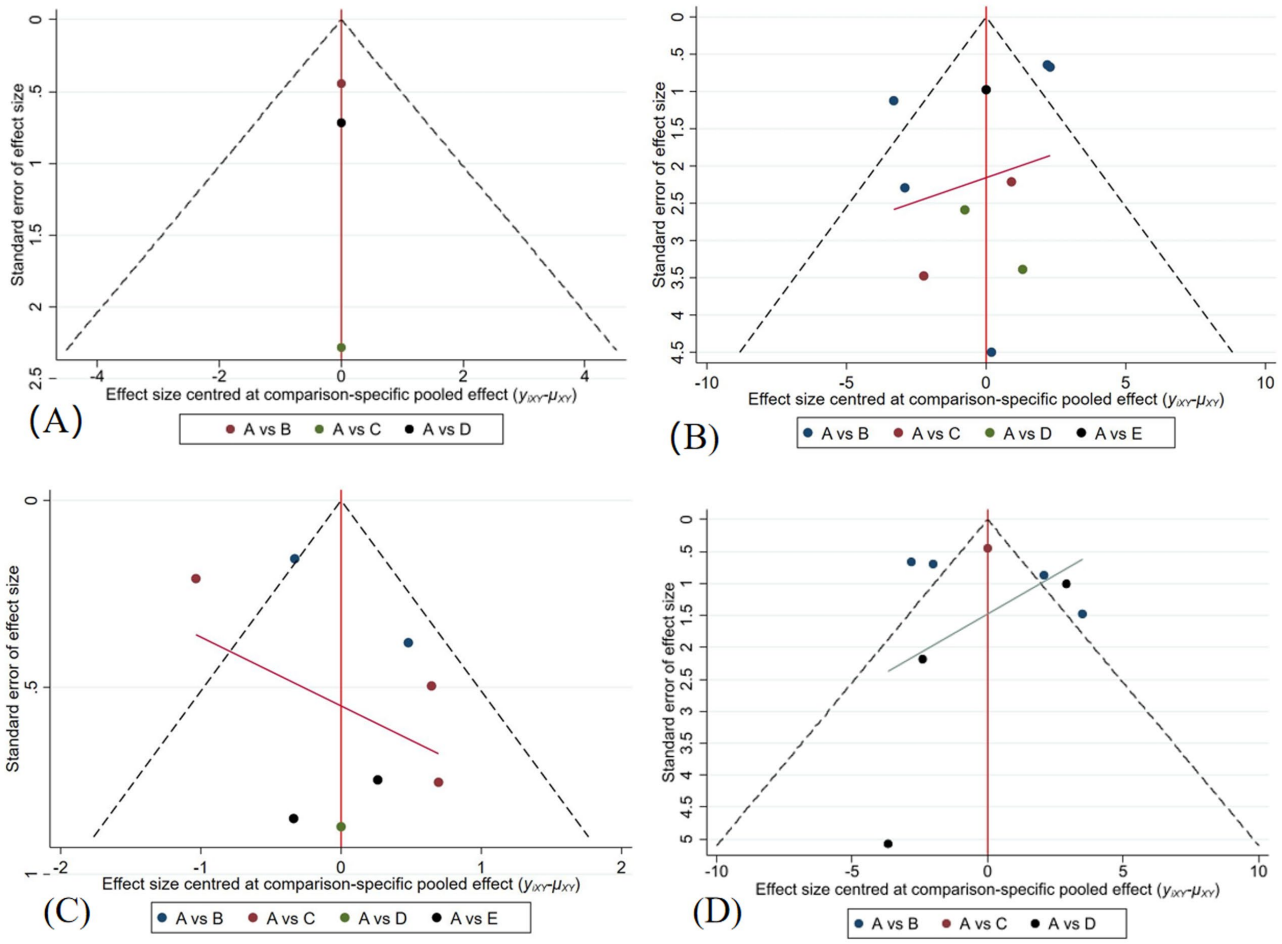
**(A)** A Funnel Plot for BBS Scores in a Healthy Older Adults; **(B)** A Funnel Plot for BBS Scores in the Patients with Parkinson’s Disease; **(C)** A Funnel Plot for TUG Times in a Healthy Older Adults; **(D)** A Funnel Plot for TUG Times in the Patients with Parkinson’s Disease.

Funnel plots were employed to assess publication bias within the included study outcome measures, specifically BBS scores and TUG time. Each study was positioned with the midline serving as the symmetry axis, with the majority of studies clustering around this axis and exhibiting rough symmetry on both sides, suggestive of minimal publication bias. However, a few isolated points were observed to be dispersed beyond the symmetry axis, potentially indicating the presence of small sample size effects (Figure 15).

## Discussion

Approximately 30 to 40% of community-dwelling older adults aged 65 and above experience falls annually, with women facing a higher risk than men (58). Fall prevention is paramount in mitigating the decline of functional capacity in this population (59). Exercise interventions, including dancing, have been shown to enhance balance abilities, strengthen muscles, improve lower limb flexibility, diminish fall risks, and alleviate fall-related anxiety (60). Dancing, as a joyful and engaging physical activity (61), may offer more sustained benefits than traditional balance and strength training.

Meta-analytic evidence underscores the positive impact of dance interventions on static balance (assessed by BBS scores and SLS time), dynamic balance, and mobility (measured by TUG time) (17), all of which are crucial factors influencing fall risk in older adults (62). For older individuals with chronic conditions, especially with Parkinson’s disease, dance practice not only improves balance but also alleviates symptoms and enhances quality of life (63).

However, there is a dearth of consensus on the “optimal” dance intervention for minimizing fall risks in older adults. The studies included in this paper primarily assessed fall risk through TUG time, Tinetti score, BBS score and SLS time (49). By conducting both direct and indirect comparisons, we aimed to evaluate the efficacy of various dance interventions in reducing fall risks among older adults, thereby facilitating the selection of the most effective dance intervention.

Age-related alterations in the sensorimotor and neuromuscular systems adversely impact the performance of both static and dynamic postural control among older adults (64). To assess these changes, BBS is employed, which evaluates both dynamic and static balance through a comprehensive set of 14 mobility-related tasks categorized into three domains: sitting balance, standing balance, and dynamic balance (65). Berg Balance Score (BBS) was significantly improved in the creative dance group compared to the control group (*p* < 0.05). Additionally, Creative dance was the most effective intervention. Research indicates that training to respond to the rhythms of improvisational music can lead to the development of highly automated gait patterns, resulting in enhanced gait performance and improved balance and mobility (66). Creative dance, which requires participants to adapt improvisationally to changes in music such as rhythm, tempo, and melody, fosters continuous reactions to external stimuli within a dynamic spatio-temporal environment (67). This type of dynamic training (68) has been shown to be beneficial for improving physical factors associated with falls, including strength, dynamic balance, and mobility, thereby contributing to fall prevention (42).

The Timed Up and Go (TUG) time offers an observational assessment of gait patterns and indirectly quantifies critical fall risk factors, including muscle strength, balance, and gait stability among individuals (28). The network meta-analysis conducted on the Timed Up and Go (TUG) time revealed that among healthy older adult, interventions involving Folk dance, Creative dance, and Ballroom dance were all significantly more effective than the control group in improving performance. Notably, Folk dance emerged as the most efficacious intervention among the three dance styles evaluated.

Slow-paced dance and various support base patterns are pivotal factors in enhancing posture and balance adjustments. Folk dance training, which involves rhythmic motor coordination and control of movements or postures, contributes to the improvement of three bodily systems: the central nervous system, sensory nervous system, and muscular system (69). This type of training benefits the core muscles associated with balance capabilities (70). Muscle strength is a fundamental component for maintaining body balance during movement. Slow-motion exercises can effectively stimulate the functionality of core muscles. Traditional Srichiangmai dance represents an alternative form of aerobic exercise characterized by its slow tempo and low impact. By adhering to the musical rhythm, it engages muscles in various body parts to sustain dance poses, thereby aiding in the enhancement of muscle strength and the improvement of balance (71). Greek traditional dance often requires prolonged single-leg standing, a posture that strengthens the role of the ankle joint in static postural control, effectively reducing postural sway in older adults and significantly increasing single-leg standing time (72). Additionally, the diverse activities involved in traditional Greek dance, including head and body rotations, weight shifts, and transitions from double-leg to single-leg stances, continually challenge the postural control system. These movements have been shown to improve specific aspects of dynamic balance control (73). Thai classical dance is characterized by its slow, continuous dance and musical rhythms. The combination of its movements and rhythms is comparable to Tai Chi, and it also features intricate postures and coordination patterns. Thai classical dance training programs consist of various modes of stretching and repetitive movements, involving the head, upper body, and the entire body’s center of gravity on various support bases. Despite differing stimulation modes of sensory information, these activities necessitate adjustments to the postural system to maintain control over posture and body orientation, potentially influencing the dancer’s balance performance (74). Furthermore, folk dances are often associated with specific cultural backgrounds. Older adults participating in such dances may experience increased engagement and enjoyment due to cultural identity and social interaction, leading to additional physical and psychological benefits. Additionally, the popularity of folk dances among communities and older populations may provide greater access and opportunities for older adults to engage in these dances, thereby benefiting from their fall risk reduction effects.

The Network meta-analysis conducted using the Berg Balance Scale (BBS) revealed that, among patients with Parkinson’s disease, interventions involving Folk dance and Ballroom dance were both significantly more effective than the control group in improving performance. Additionally, Folk dance exhibited a superior intervention effect compared to Social dance.

The optimal exercise intervention for individuals with Parkinson’s disease (PD) should integrate cueing strategies, balance training, focused attention, and enhancement of physical fitness (75). Traditional folk dances have the capacity to stimulate specific deep limbic neuronal circuits, eliciting emotional engagement while incorporating movement elements within the rhythmic structure of the dance, which can be understood as a therapeutic modality (76). For instance, Irish Set Dancing has gained popularity among PD patients due to its relative simplicity and ease of learning and execution (39). This dance form combines dynamic balance activities with gait and skilled movements (39), leading to improvements in PD motor symptoms such as balance impairments, freezing of gait, and bradykinesia (75, 77, 78). Furthermore, Irish dance incorporates elements of exercise, socialization, and partner involvement, motivating PD patients to adhere to long-term physical activity (39). This integrated approach to Folk dance not only addresses the motor symptoms of PD but also fosters emotional well-being and social engagement, which are crucial components of patient care.

In older adult with Parkinson’s disease (PD), specific risk factors render them more susceptible to falls and fall-related injuries. Tango is a kind of social dance with the potential to mitigate balance impairments in this patient population, thereby aligning with previous meta-analytic findings that highlight its benefits (75, 79, 80). Argentine Tango, in particular, may enhance motor capabilities by targeting PD-related impairments such as spontaneous changes in movement direction/speed and frequent transitions between movement initiation and cessation. These features could specifically address difficulties in movement initiation, turning, and bradykinesia. For instance, patients with PD often exhibit slowed movements, but with the aid of external cues, they can achieve near-normal speed and amplitude of movements (77, 81). Such external cues may access cortical circuits, bypassing the dysfunctional basal ganglia in PD patients (82). Ballroom dance, particularly its social aspect, offers crucial external cues: music and a dance partner. Music serves as an auditory cue, enhancing gait speed, initiation speed, and step frequency when utilized. A partner, through physical contact, can further augment balance, as even mild touch can facilitate postural stability (83). The connection between partners may aid in movement initiation and increase or maintain step length and rhythm (84). While physical and psychological benefits accrue to PD patients engaging in exercise training, the severity of the disease influences motor responsiveness; thus, this should be considered when selecting intervention modalities. Only in patients with milder symptoms and slower disease progression (85) does exercise appear to reduce fall incidents.

Study Limitations: (1) The limited number of studies and modest sample sizes incorporated in this analysis may introduce bias into the study outcomes. To mitigate this, we recommend conducting a large-scale, multi-arm randomized controlled trial (RCT) specifically focused on dance interventions, thereby enabling a more robust evaluation of the effectiveness of individual dance types in preventing falls among older adults. (2) The heterogeneity in the fall indicators assessed across the included studies precludes the analysis of more generalized metrics. To address this, we suggest adopting standardized testing methodologies that comprehensively assess fall risk in older adults, thereby facilitating cross-study comparisons and enhancing the reliability of findings. (3) The research focused solely on the analysis of healthy older adult and patients with Parkinson’s disease, and did not comprehensively examine other chronic disease patient groups. Consequently, we propose that future dance interventions targeting patients with chronic diseases should be implemented, with careful consideration given to the potential moderating effects of age, gender, and disease severity on motor function outcomes.

## Conclusion

Creative dance, Folk dance, and Ballroom dance effectively reduce the risk of falls in older adults. Creative dance enhanced the Berg Balance Score the most among healthy older adults, while Folk dance improved the Timed Up and Go test performance the best. Similarly, among patients with Parkinson’s disease, Folk dance exhibits the best effect in improving Berg Balance Scale scores. Future research should incorporate more high-quality randomized controlled trials (RCTs) to elucidate the effectiveness of different dance styles in mitigating falls among older adult with varying health statuses, thereby addressing the unique needs of diverse older adult for fall prevention.

## Data Availability

The original contributions presented in the study are included in the article/supplementary material, further inquiries can be directed to the corresponding author.
